# A Model of a MAPK•Substrate Complex in an Active Conformation: A Computational and Experimental Approach

**DOI:** 10.1371/journal.pone.0018594

**Published:** 2011-04-11

**Authors:** Sunbae Lee, Mangalika Warthaka, Chunli Yan, Tamer S. Kaoud, Andrea Piserchio, Ranajeet Ghose, Pengyu Ren, Kevin N. Dalby

**Affiliations:** 1 Division of Medicinal Chemistry, University of Texas at Austin, Austin, Texas, United States of America; 2 Department of Biomedical Engineering, University of Texas at Austin, Austin, Texas, United States of America; 3 Graduate Program in Pharmacy, University of Texas at Austin, Austin, Texas, United States of America; 4 Department of Chemistry, The City College of New York, New York, New York, United States of America; 5 The Graduate Center of The City University of New York, New York, New York, United States of America; 6 Graduate Program in Biomedical Engineering, University of Texas at Austin, Austin, Texas, United States of America; 7 Graduate Program in Biochemistry, University of Texas at Austin, Austin, Texas, United States of America; University of South Florida College of Medicine, United States of America

## Abstract

The mechanisms by which MAP kinases recognize and phosphorylate substrates are not completely understood. Efforts to understand the mechanisms have been compromised by the lack of MAPK-substrate structures. While MAPK-substrate docking is well established as a viable mechanism for bringing MAPKs and substrates into close proximity the molecular details of how such docking promotes phosphorylation is an unresolved issue. In the present study computer modeling approaches, with restraints derived from experimentally known interactions, were used to predict how the *N*-terminus of Ets-1 associates with ERK2. Interestingly, the *N*-terminus does not contain a consensus-docking site ((R/K)_2-3_-X_2-6_-Φ_A_-X-Φ_B_, where Φ is aliphatic hydrophobic) for ERK2. The modeling predicts that the *N-*terminus of Ets-1 makes important contributions to the stabilization of the complex, but remains largely disordered. The computer-generated model was used to guide mutagenesis experiments, which support the notion that Leu-11 and possibly Ile-13 and Ile-14 of Ets-1 *1-138 (Ets)* make contributions through binding to the hydrophobic groove of the ERK2 D-recruiting site (DRS). Based on the modeling, a consensus-docking site was introduced through the introduction of an arginine at residue 7, to give the consensus ^7^RK-X_2_-Φ_A_-X-Φ_B_^13^. This results in a 2-fold increase in *k*_cat_/*K*_m_ for the phosphorylation of *Ets* by ERK2. Similarly, the substitution of the *N*-terminus for two different consensus docking sites derived from Elk-1 and MKK1 also improves *k*_cat_/*K*_m_ by two-fold compared to *Ets*. Disruption of the *N*-terminal docking through deletion of residues 1-23 of *Ets* results in a 14-fold decrease in *k*_cat_/*K*_m_, with little apparent change in *k*_cat_. A peptide that binds to the DRS of ERK2 affects *K*_m_, but not *k*_cat_. Our kinetic analysis suggests that the unstructured *N*-terminus provides 10-fold uniform stabilization of the ground state ERK2•*Ets•MgATP* complex and intermediates of the enzymatic reaction.

## Introduction

Mitogen-activated protein kinases (MAPKs) are cell-signaling enzymes that regulate an extraordinarily diverse range of biological processes in eukaryotic organisms [1], [2]. However, despite this diversity of signaling, MAPKs are characterized by a single pronounced specificity, namely a preference for phosphorylating proteins at a Ser/Thr-Pro motif [3]. This specificity comes from their ability to negatively select against many potential substrates by virtue of the activation segment, a loop at the active site that creates a shallow hydrophobic pocket most compatible with the binding of proline [3], [4]. Despite this specificity, proteins containing just a Ser/Thr-Pro element are poor MAPK substrates, generally exhibiting large Michaelis-Menton constants when compared to specific protein substrates. This difference in specificity reflects the weak interaction of the Ser/Thr-Pro motif with the MAPK active site [5] and the importance of docking interactions. Docking interactions underlie the ability of some MAPKs to phosphorylate as many as fifty substrates or more *in vivo* [6].

**Figure 1:**
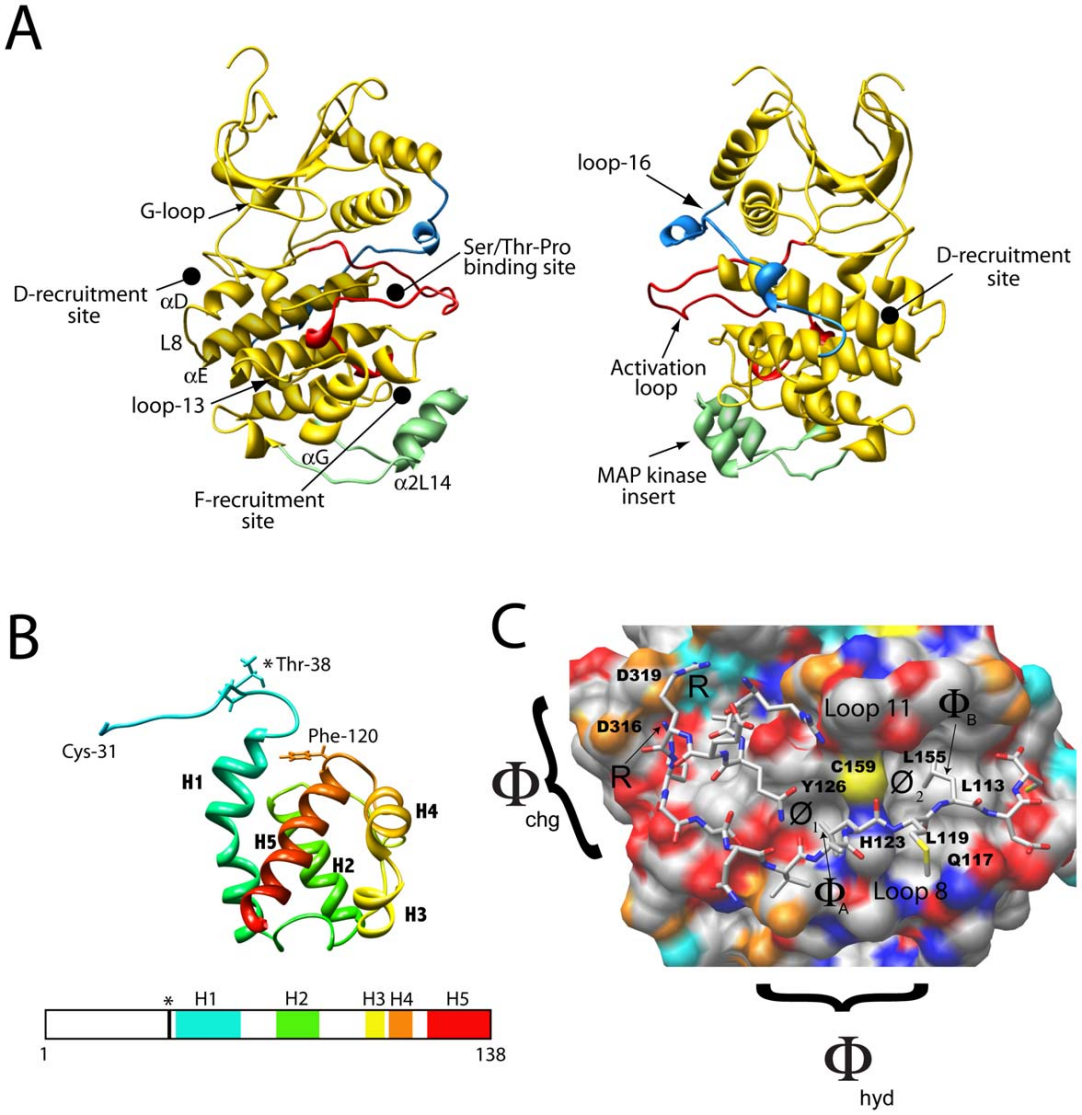
Structural Features of ERK2 and Ets-1. **A**) Ribbon diagrams of activated ERK2 (PDB: 2ERK) showing the G-loop, which clamps onto ATP, and the binding site for substrate Ser/Thr-Pro motifs (which become phosphorylated). Also indicated are the D and F recruitment sites, and loop-16 (blue), which communicates between the D-recruitment site and the activation loop (red). A small insert, unique to MAP kinases, called the MAPK insert is also shown (*colored green*, residues 246–276). The *D-recruitment site* is comprised of the reverse turn (Asn-156–Asp-160) between the β7 sheet and the β8 sheet, part of loop 7 (Glu-107–Asp-109), the αD helix (Leu-110—Thr-116), loop 8 (Gln-117–Ser-120) and part of the αE helix (Asn-121–Phe-127) and the *Common Docking (CD) domain* (Asp-316 and Asp-319). The *F-recruitment site (indicated)* is a hydrophobic pocket with a preference for binding a Ψ-X-Ψ motif (where ψ are aromatic residues). The pocket is comprised of the *C*-terminus of the activation loop starting at Phe-181 through to the end of loop 12 (Phe-181–Thr-204), the *α*G helix (Tyr-231–Leu-242), and the *a*2L14 of the MAPK insert helix (Leu-256–Leu-263). **B**) Ribbon representation of residues 31-138 of Ets-1 (PDB ID: 2JV3). The ERK2 phosphorylation site, Thr-38, is shown as well as Phe-120 a residue implicated in ERK2 binding. The five helices, H1-H5, that comprise the SAM domain of Ets-1 are shown. **C**) Binding of the D-site peptide RLQERRGSNVALMLDV (consensus; (∑)_2-3_-X_4-6_-Φ_A_-X-Φ_B_, where ∑ are basic residues and Φ are large hydrophobic residues), derived from hematopoietic protein tyrosine phosphatase, to unactivated ERK2. The Φ_A_ and Φ_B_ residues of hematopoietic protein tyrosine phosphatase occupy the Ø_1_ and Ø_2_ pockets of the Φ_hyd_ subsite, respectively. The Φ_chg_ subsite of the DRS is indicated. Surface representations; red, oxygen; orange, carboxylate oxygen; blue, neutral nitrogen; cyan, ε lysine nitrogen, or arginine guanidino nitrogen.

MAPK docking interactions are mediated through MAPK recruitment sites, which are known to recognize modular-docking sequences called docking sites [7]. When these recruitment sites bind canonical substrate docking sites they are thought to increase the effective concentration of the substrate Ser/Thr-Pro motif near the active site, and thereby increase the rate of substrate turnover [5]. For example, Extracellular signal-regulated kinase (ERK2) has two recruitment sites called the D-recruitment site (DRS) and the F-recruitment site (FRS) that bind D-sites and F-sites respectively [8]. A D-site is a modular motif with a basic and hydrophobic composition, (∑)_2-3_-X_2-6_-Φ_A_-X-Φ_B_, where ∑ is basic and Φ is aliphatic hydrophobic. In contrast, the F-site is a smaller hydrophobic motif, ΨXΨP, where Ψ is aromatic [9], [10]. The DRS is an extended peptide-binding groove found on the rear face of the MAPK (A negatively charged surface (Φ_chg_), containing two common-docking Asp residues and a nearby hydrophobic docking groove (Φ_hyd_) constitute the D-recruitment site (DRS) of ERK2), while the FRS is a relatively shallow hydrophobic pocket found adjacent to the Ser/Thr-Pro binding site. Fig. 1A shows the general structural organization of ERK2 and the positions of the DRS and FRS relative to the active site.

**Figure 2:**
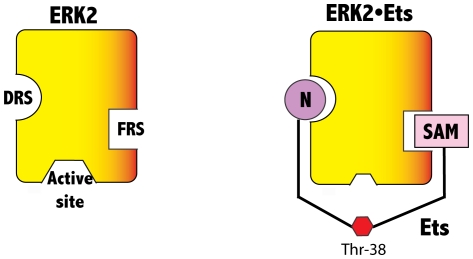
Schematic representation of ERK2, and the ERK2•*Ets* complexes depicting the DRS, the FRS and the active site. The *N-*terminal docking site and the SAM domain of *Ets* are shown binding to the DRS and the FRS of ERK2 respectively. The phosphorylation site of *Ets,* Thr-38 is depicted in proximity to the active site.

Precisely how these recruitment sites facilitate the phosphorylation of a substrate has not been established, because no structure of a substrate bound to a MAPK has been reported. Thus, key questions regarding the mechanism of substrate phosphorylation include understanding how docking interactions control the rate of access of phosphorylation sites to the active site, what steps control and limit catalysis and what, if any general features substrates possess. To address these questions we have examined the mechanism of phosphorylation of the transcription factor Ets-1 by ERK2 (See Fig. 1B for the structure of residues 29–138 of Ets-1). Ets-1 is phosphorylated by ERK2 with high specificity, and is intriguing because it uses two docking sites to bind to ERK2 [11], [12], [13], neither of which appear to correspond to a canonical modular sequence. Seidel et al. originally located one docking site to a five-helix bundle called the *Pointed* domain (more generally a sterile alpha motif (*SAM*) domain) [14]. We recently located a second site in the intrinsically disordered *N*-terminus and proposed a model for the interaction between ERK2 and Ets-1 where the SAM domain and the *N*-terminus interact with the FRS and the DRS of ERK2, respectively (Fig. 2) [11], [12], [13]. A feature of this model is that it places the phosphorylation site in the proximity of the active site.

In the present study we use a computational approach complimented by steady-state kinetic experiments to extend our understanding further to elucidate a more detailed model for the binding of residues 1–138 of Ets-1 (*Ets)* to ERK2. The modeling suggests that the *N*-terminus remains disordered, with residues 10–16 loosely associating with the hydrophobic groove of the DRS. A putative Φ_A_-X-Φ_B_ motif is identified comprised of ^11^LTI^13^. Our kinetic analysis suggests that the unstructured *N*-terminus provides 10-fold uniform stabilization of the ground state ERK2•*Ets•MgATP* complex and intermediates of the enzymatic reaction. To our knowledge, this represents the first model for the structure of an activated MAPK•substrate complex.

## Materials and Methods

### Reagents

NovaSyn TGR resin was purchased from Novabiochem (Gibbstown, NJ). Fmoc-6-aminohexanoic acid was purchased from AnaSpec (Fremont, CA). Other Fmoc-amino acids, HBTU, and HOBT were obtained from Advanced ChemTech (Louisville, KY). Ultrapure grade Tris and HEPES were obtained from Sigma (St. Louis, MO). MP Biomedicals (Irvine, CA) supplied [*γ*-^32^P]-ATP. P81 Ion Exchange Cellulose Chromatography Paper was purchased from Whatman (Piscataway, NJ). Yeast extract, tryptone, agar, and IPTG were obtained from US Biologicals (Swampscott, MA). Ni-NTA agarose, Quiaprep Spin miniprep Kit, PCR QIAquick Purification Kit, and QIAEX II Gel Extraction Kit were supplied by Qiagen Inc. (Valencia, CA). Restriction enzymes, PCR reagents, and T4 DNA ligase were purchased from New England Biolabs (Beverly, MA) and Invitrogen Corp. (Carlsbad, CA). Oligonucleotides were purchased from Sigma. A Mono Q HR 10/10 anion exchange column was purchased from Amersham Biosciences (Piscataway, NJ). The *Escherichia coli* strain DH5α, used for cloning and mutagenesis, and the strains BL21 (DE3) used for recombinant protein expression, were obtained from Invitrogen. The pET28a vector was purchased from Novagen. The remaining molecular biology reagents, including DNA ladders and protein molecular mass standards, were obtained from Invitrogen Corp. All other buffer components and chemicals were obtained from Sigma.

#### Peptide Synthesis and Purification

Peptides were synthesized on rink resin (NovaSyn TGR resin) using a solid phase peptide synthesizer (Liberty CEM Automated Microwave Peptide Synthesizer, or a Rainin Quartet Peptide Synthesizer by utilizing an Fmoc solid-state peptide synthesis protocol. Synthesized peptides were acetylated at the *N*-terminus and then cleaved using a cleavage cocktail (1 mL thioanisole, 0.5 mL H_2_O, 0.5 mL ethanedithiol, and 18 mL trifluoroacetic acid). The cleaved peptides were precipitated with 45 mL ethyl ether. After centrifugation the ethyl ether was removed and the precipitated peptides resuspended in 15% aqueous acetonitrile and freeze-dried. Crude peptides were purified by HPLC using a reverse phase C-18 column (Hi-Pore RP-318, Bio-Rad) on an AKTA system (Amersham) using a gradient of H_2_0 (0.1% trifluoroacetic acid) against acetonitrile (0.1% trifluoroacetic acid) with a flow rate of 2 mL/min. The peptides were subjected to an elution profile of 0–10% acetonitrile 0–5 min; 10–30% 5–60 min. Purified peptides were characterized for purity and mass by analytical HPLC (System Gold, Beckman Coulter) followed by mass spectrometry using either a MALDI-TOF (Voyager, PerSeptive Biosystem) or an ESI (LCQ, Thermo Finnigan). The following analytical results were obtained; Lig-F, observed 1860.01, calculated mass 1860.99; Lig-D, observed 2209.98, calculated mass 2211.31).

### Molecular Biology

A pET-28a bacterial expression vector encoding a hexa-histidine tag followed by the cDNA encoding murine Ets-1 residues 1-138 (pET-28a *Ets*, a gift of L. P. McIntosh, University of British Columbia, Vancouver) was modified by PCR using site directed mutagenesis to construct an *N*-terminal truncation mutant containing Ets-1 residues 24-138 (Δ23N-*Ets*) with an initial methionine (pET-28a ΔN23Ets).

#### Construction of pET-28a ΔN23-Ets N-terminal truncation mutant

pET-28a *Ets* was PCR amplified with a forward primer containing an NdeI site (encoding the initial methionine) followed by the codon encoding Phe-24 (5′-GG GAA TTC CAT ATG TTC CCT TCC CCG GAC ATG-3′) and an outer reverse primer (5′-GCT AGT TAT TGC TCA GCG GTG G-3′) using the following PCR conditions: 94°C for 5 min to denature the complementary strands; 30 cycles of 55°C for 30 sec to anneal the primers, extension for 1 min at 72°C, followed by a denaturation step at 94°C for 45 sec; complementary strands were extended a final 10 min at 72°C. The *N*-terminal mutant PCR product was digested with NdeI and HindIII and ligated into NdeI-HindIII digested pET-28a. All proteins produced from pET-28a have an *N*-terminal sequence of M-G-S-S-H-H-H-H-H-H-S-S-G-L-V-P-R-G-S-H- prior to the initial methionine encoded by the Ets cDNA giving EtsΔ24-138 an approximate mass of 15, 391 Da whereas Ets 1-138 has a mass of 17, 681 Da (lacking the initial methionine).

#### Construction of mutants of single cysteine - (Cys-31) *Ets*

pET28a-*Ets* was modified by overlap extension polymerase chain reaction to construct the single cysteine mutant of *Ets* (*C99A/C106A/C112A)* in an *Ets* S26A background as reported elsewhere [15]. Mutations were produced by a single-step PCR reaction using the following PCR amplification reaction (50 µL) contained Phusion DNA polymerase GC buffer, 200 µM each of the four deoxynucleoside triphosphates, 2 mM MgCl_2_, 3% DMSO, 80 ng of template DNA (Ets-C31 pET28a vector), 0.5 µM primers forward and reverse, and 1 µL of Phusion DNA polymerase (Finnzymes, USA, Product # F-530-S). The cycling parameters were 98°C for 2 min, followed by 35 cycles at 98°C for 10 s, 72°C for 30 s, with a final elongation step 72°C for 10 min. Forward primers (I13A/I14A: 5- **ATG GCT AGC** ATG AAG GCG GCC GTC GAT CTC AAG CCG ACT CTC ACC **GCA GCA** AAG ACA GAA-3, I13D/I14D: 5-**ATG GCT AGC** ATG AAG GCG GCC GTC GAT CTC AAG CCG ACT CTC ACC **GAC GAC** AAG ACA GAA-3, L11D: **ATG GCT AGC** ATG AAG GCG GCC GTC GAT CTC AAG CCG ACT **GAC** ACC ATC ATC AAG, I13D: **ATG GCT AGC** ATG AAG GCG GCC GTC GAT CTC AAG CCG ACT CTC ACC **GAC** ATC AAG ACA GAA, L7R: **ATG GCT AGC** ATG AAG GCG GCC GTC GAT **CGA** AAG CCG ACT CTC, D6R/L7R: **ATG GCT AGC** ATG AAG GCG GCC GTC **CGA CGA** AAG CCG ACT CTC, Elk-1312-324: **ATG GCT AGC** ATG AAG GCG **AAA** **GGC CGC AAA CCG CGC GAC CTG GAA CTG CCG** AAG ACA GAA AAA GTG GAT CTC GAG C, MKK1 1-13: **ATG GCT AGC** ATG AAG **ATG CCG AAA AAA AAA CCG ACC CCG ATT CAG** **CTG AAC** AAG ACA GAA AAA GTG GAT CTC GAG C) (*Nhe*I site and mutations are in bold) and reverse primer (5-CAG AAA GAG GAT GTG AAA TAA **CAA GCT TGC** -3) (*Hin*dIII site in bold) were used to generate the mutants. The PCR product was digested with *Nhe*I and *Hin*dIII, ligated into the *Nhe*I-*Hin*dIII digested pET28a vector and then transformed into DH5α *E. coli* cells. The construct was verified by sequencing the DNA at the UT core facilities

#### Preparation of Proteins

Activated tagless ERK2 was generated essentially as described in (Kaoud, T.S. et al. manuscript in preparation). Expression and purification of Ets-1 (1-138) was followed by the method described in the previously published literature [15].

### Data Analysis

#### Steady-state kinetic experiments

zReactions were carried out at 27°C in kinase assay buffer (25 mM HEPES pH 7.4, 100 mM KCl, 2 mM DTT, 40 µg/mL BSA, and 20 mM MgCl_2_) containing 2 nM ERK2 and varied concentrations of substrates and inhibitors. Rates were measured under conditions where total product formation represented less than 10% of the initial substrate concentrations. The reaction was incubated for 10 min before initiation by addition of enzyme and quantified as described previously [16]. Initial rates were determined by linear least squares fitting to plots of product against time. Reciprocal plots of 1/*v* against 1/*s* were checked for linearity, before the data were fitted to eqn. 1 using a non-linear least squares approach, assuming equal variance for velocities, using the program Kaleidagraph 3.5 (Synergy software). The intercepts 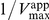 and slopes 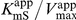 obtained from these fits were then plotted against either the inhibitor concentration (*i*) (for inhibition experiments) or the reciprocal of the non-varied substrate concentration (1/*s*) (for initial velocity experiments). These plots were used to determine the appearance of the overall kinetic equation. Values for kinetic constants were then obtained using the program Scientist (Micromath) by fitting the kinetic data to the relevant over-all equation. Data conforming to a sequential initial velocity pattern were fitted to eqn 2; data conforming to linear competitive inhibition were fitted to eqn. 3; data conforming to hyperbolic mixed inhibition were fitted to eqn. 4. Dose-response curves for data conforming to linear inhibition were fitted to eqn. 5; and for data conforming to hyperbolic inhibition to eqn. 6.
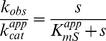
(1)

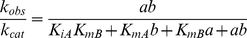
(2)

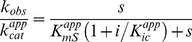
(3)

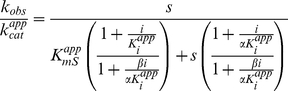
(4)

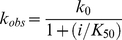
(5)

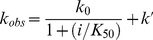
(6)


The parameters used in deriving equations are defined as follows; 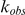, observed rate constant; 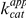, apparent catalytic constant; *s*, concentration of substrate S; 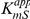, apparent Michaelis constant for substrate S; *a*, concentration of substrate A; *b*, concentration of substrate B; 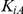, inhibition constant for substrate A; 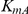, Michaelis constant for substrate A; 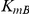, Michaelis constant for substrate B; *i*, concentration of inhibitor *I*; 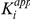 or 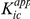, apparent competitive inhibition constant for inhibitor *I*; 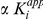, apparent uncompetitive inhibition constant for inhibitor *I*; 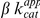, apparent catalytic constant for enzyme inhibitor complex; 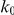, is the observed rate constant in the absence of inhibitor, 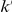 is the observed rate constant at saturating inhibitor, *I*, or activator *x*, 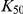 is the concentration that leads to half the maximal change in 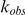.

### Molecular Modeling

#### Construction of models of peptide ligands

To facilitate the virtual docking of peptides to ERK2, we constructed the initial peptide structures based on homology using Modeler [17] when possible. This step, while not absolutely necessary, likely improves the search for optimal docked structures. Here we briefly describe the approach used to model the structures of three peptide sequences onto the surface of activated ERK2:

- For sequence *FQRKTLQ-* ***RRNLKGLNLNL*** (Lig-D), coordinates were first obtained for the fragment RRNLKGLNLNL using the known structure of this fragment bound to the MAPK FUS3 (PDB ID: 2B9H) [18]. Next, the remaining seven residues FQRKTLQ were added to the *N*-terminus using homology software (Modeller9v4) [17]. By using “−−−−−−−RRNLKGLNLNL” as the template and “FQRKTLQRRNLKGLNLNL” as the target, Modeller predicts the possible 3-D structures for the seven residues based on the sequence similarity to a library of loops with known structures. Ten structures were produced, and the one with the best score was used for subsequent virtual docking.
- *YAPRAPAKLA* ***FQFP*** *SR* (Lig-F) - coordinates were first obtained for the FSFG motif from mRNA export factor MEX67 (PDB ID: 2KHH) [19]. The FSFG motif was then transformed to FQFP using the LEaP module [20] of Amber 9.0, with the new side chains placed randomly. The remaining residues were modeled as in a).
- Residues 1-28 of Ets-1 were modeled onto Ets-1 (residue 29-138) (PDB ID: 2JV3) [21] using Modeller as described in a). In this case, five structures were generated. The residues 1-42 of the five structures were subsequently used in docking to ERK2.

#### Defining binding pockets and restraints

a) Binding within the DRS – The crystal structure of a docking site peptide (RLQERRGSNVALMLDC) derived from the protein phosphatase HePTP in complex with inactive ERK2 (PDBID: 2GPH) [22] was used to define the binding pocket of Lig-D within the DRS of ERK2. All residues on ERK2 within 20 Å from the peptide were selected as the constituents of the DRS pocket. During computational docking, distance constraints between the peptide FQRKTLQ**RRNLKGLNLNL (Lig-D)** and ERK2 were set according to the known interactions of the two motifs (RR and Φ_A_-X-Φ_B_) with the phosphatase. The structure of 2GPH indicates that Φ_B_ in the Φ_A_-X-Φ_B_ motif will make Van der Waals contacts with Leu-113 of αD in ERK2, Phe-127 of αE, Leu-155 of *β*7, Cys-159 in the *β*7-*β*8 loop, and Thr-108 in the crossover connection. Φ_A_ binds to a hydrophobic patch on the surface of helix αE in ERK2, formed by His-123 and Tyr-126, and Cys-159 in the *β*7-*β*8 hairpin. Asp-316 and Asp-319 in the CD site of ERK2 can form direct contacts with the RR motif [22]. b) Binding within the FRS – Previous studies have shown that a docking site motif containing the Phe-Xaa-Phe motif (DEF motif, or F-site) binds a distinct hydrophobic pocket formed between the P+1 site, the αF helix and the MAP kinase insert [23]. Thus, residues, Leu-144–Leu-153, Phe-181–Asn-199, and Phe-226–Leu-262, were assigned as binding pocket residues for the F-site of Lig-F (YAPRAPAKLA**FQFP**SR). These interactions were also utilized as loose constraints in the docking.

### Computational docking

Computational docking studies were performed with GOLD 4.1 (The Cambridge Crystallographic Data Centre [24], [25]). The x-ray coordinates of the active form of the MAP kinase ERK2 (PDBID: 2ERK) [3] were used to dock each of Lig-D and Lig-F. Distance constraints were applied between certain peptide residues and ERK2 to guide the docking, and the tolerance is typically +/− 1 Angstrom. Specific information about the distance constraints, including atom pairs, force constants and the distance ranges are listed in Table S1. During the docking, the protein was kept rigid and peptides were flexible. In each run, 2,500,000 genetic operations were performed on an initial population of 200 members, which were divided into five sub-populations. The default parameters were used for the rest of the settings. A total of 50 structures were generated at the end of each docking run. ChemScore [24] was used to rank the predicted structures.

#### Modeling of ERK2-Ets-1 complex

The fragment (residues) 1-42 docking to ERK2 were predicted using GOLD 4.1 (The Cambridge Crystallographic Data Centre [24], [25], again with the TP residue constrained in the active site. As described earlier Modeller was used to generate 5 initial structures for this fragment. Since the peptide is longer than the other peptide substrates, the search for optimal binding mode is very challenging. To achieve confident results, all five structures were used in five independent docking runs. The complex structure of active ERK2 (PDB ID: 2ERK) bound to Ets-1 (residues 29–138) (PDB ID: 2JV3) [21] was predicted using the molecular mechanics modeling approaches using a semi-rigid body potential smoothing energy minimization as described previously [13]. To assemble a model for complete Ets-1 (1–138) complexed to ERK2, the two models ERK2•Ets(1–42) and ERK2•Ets(29–138) were aligned and a new model ERK2•Ets-1 (1–138) derived by retaining residues 1–39 and 40–138 from the two models, respectively.

## Results

### Modeling the ERK2•Ets-1 complex

A considerable amount of experimental data suggests that activated ERK2, is a monomer *in vitro*, and forms a 1:1 complex with residues 1–138 of Ets-1 where two docking interactions contribute to the stability of the complex [11] (Fig. 2). The SAM domain of Ets-1 mediates one of the docking interactions, while the second involves its intrinsically disordered *N*-terminus [13]. Recently, using a molecular dynamics approach, we built a model (Model A) for the complex formed between residues 29–138 of Ets-1 and ERK2, which reveals how the SAM domain may bind ERK2 [13]. However, this model does not address the role of the disordered *N*-terminus of Ets-1. To address how the *N*-terminus binds ERK2 we have used a combination of computational and experimental approaches to define the general topology of the ERK2•*Ets* complex and to identify a possible catalytic function for the docking interaction.

**Figure 3:**
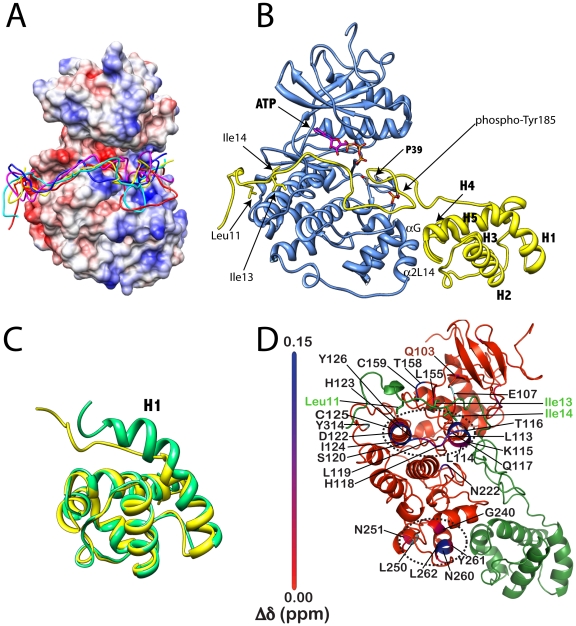
Model of ERK2•*Ets* Complex. **A**) Modeling Residues 1–42 of Ets-1 onto the Surface of ERK2 showing four of the top five ranked solutions binding to the DRS of ERK2. Coulombic surface representation was performed in Chimera using default parameters. **B**) A model of the activated ERK2•*Ets* complex determined by molecular modeling (See Experimental Procedures). The ATP molecule is superimposed on the structure following alignment with PKA (PDB ID: 1ATP). **C**) Predicted unwinding of residues 41–51 of Helix 1 upon the binding of *Ets* to ERK2. Structure of residues Ser-40−Lys-138 free (green) and predicted structure in complex (yellow) with ERK2. **D**) Chemical shift perturbations (Δδ) induced in *inactive* ERK2 in the presence of an excess of *Ets*. The perturbations are indicated using a red (smallest) to blue (largest) gradient on a ribbon representation of ERK2. Residues that display significant scaled chemical shifts (Δδ>0.08 ppm) values are labeled. Residues for which resonances that are broadened beyond the threshold of detection, in the presence of *Ets*, are colored cyan and labeled. The overall pattern of chemical shift perturbations coincides quite well with the predicted structure of the complex of *Ets* with active ERK2 with the largest Δδ values are seen near the DRS and the FRS (indicated by the dotted ovals). The gatekeeper residue (Gln-103, in red) shows a small but significant perturbation. The residues Leu-11, Ile-13 and Ile-14 from the disordered N-terminus of *Ets* (green) that are predicted to be important for the ERK2/*Ets* interaction from modeling and mutagenesis studies (see main text), are indicated. Chemical shift perturbations were calculated using a TROSY-based HNCO experiment using the following equation: 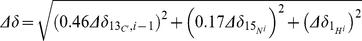
where the chemical shift changes for ^13^C’^i-1^, amide ^15^N^i^ and ^1^H^i^ nuclei are given by 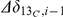, 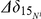 and 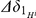 respectively. The chemical shift range has been rescaled to maximize contrast and aid visualization.

As residues 1–40 of Ets-1 are intrinsically disordered a two-step strategy was adopted to build the final model for the ERK2•*Ets* complex. First, Model B was built which describes the binding of residues 1-40 of Ets-1 to ERK2 using the docking program GOLD 4.1 (The Cambridge Crystallographic Data Centre (*8, 9*)). Following the strategy previously adopted to build Model A, the Thr-38/Pro-39 motif of *Ets* was restricted to the active site of ERK2 [13]. These restraints were imposed to reflect the orientation of the Thr-38/Pro-39 motif at the transition state for phosphoryl transfer. Under these distance restraints the remaining residues were then allowed to dock onto the surface of ERK2 (see Experimental Methods for details). The five top ranked solutions for Model B (one from each independent docking run) were found to possess similar binding modes, in which the *N*-terminus adopts a disordered conformation, with a stretch of seven residues, ^10^TLTIIKT^16^, loosely engaging the hydrophobic groove of the DRS of ERK2 (Fig. 3A). To build the final model the two models (A and B) were combined by first aligning them and then combining residues 1-39 from the best ranked Model B with residues 40-138 from Model A (Fig. 3B).

The resulting model of the ERK2•*Ets* complex represents the first structural model of a MAPK•substrate complex in an active conformation. The modeling predicts a ‘loose’ complex with a high degree of conformational flexibility. Significantly, it shows how both recruiting sites of ERK2 simultaneously engage *Ets*-1 through non-modular docking interactions to place the phosphorylation site, Thr-38, at the active site. Thus, as noted previously, the modeling predicts that the αG helix and the α2L14 helix of the FRS of ERK2 interact with the Helix 4–loop–Helix 5 motif of the SAM domain of *Ets*-1 to orient the Thr-38 towards the active site [13]. Interestingly, residues 41–51 of Helix 1 are predicted to unwind upon binding ERK2, to facilitate the formation of a new interface and to allow Thr-38 to attain proximity to the active site (Fig. 3C). What we learn from this new model is that the positioning of Thr-38 at the active site may be augmented further by the binding of residues 10–16 of the *N*-terminus of *Ets* along the hydrophobic groove of the DRS of ERK2. We also see that many residues in the *N*-terminus of *Ets* appear to play no direct role in the binding. This model explains why the phosphorylation of Ets-1 is insensitive to mutation of the common docking domain of ERK2 (e.g. D316/D319A) [11], because according to the model neither Asp-316 nor Asp-319 contribute to Ets-1 recognition. While this model must be considered to be a low-resolution approximation of the ERK2•*Ets* complex, it is nevertheless of significant value, because it provides an important framework from which to begin to understand the specificity and mechanism of ERK2 towards Ets-1 and potentially towards other substrates also. Significantly, when we examine which amino acids undergo perturbations in their NMR chemical shifts in inactive ERK2 in the presence of an excess of *Ets* we see good agreement with our model (Fig. 3D). Indeed, no chemical shift perturbations (see legend to Fig. 3D) are seen for the ERK2 common docking residues Asp-316 and Asp-319 as predicted by the model. On the other hand, significant perturbations (>0.1 ppm) were seen for several residues that constitute the hydrophobic patch on the DRS and form critical contacts with Φ_A_ and Φ_B_ residues of a canonical D-site, notably Leu-113 (0.13 ppm), His-123 (0.14 ppm), Tyr-126 (0.1 ppm) and Cys-159 (0.21 ppm). Additionally at the FRS, for residues where chemical shift assignments are available, large perturbations are seen for Asn-260 (0.17 ppm) and Leu-262 (0.14 ppm) on *a*2L14 helix of the MAPK insert. This suggests that the topology of the model is accurate with *Ets* engaging both the DRS and the FRS in both inactive and activated ERK2. A more comprehensive listing of residues that undergo significant chemical shift changes is shown in Fig. 3D. Further details about the interactions of inactive ERK2 with a range of ligands bearing canonical and non-canonical docking motifs, determined by solution NMR techniques, will be presented in details in a separate publication (Piserchio et. al. *submitted*).

### Assessing the role of the FRS

**Figure 4:**
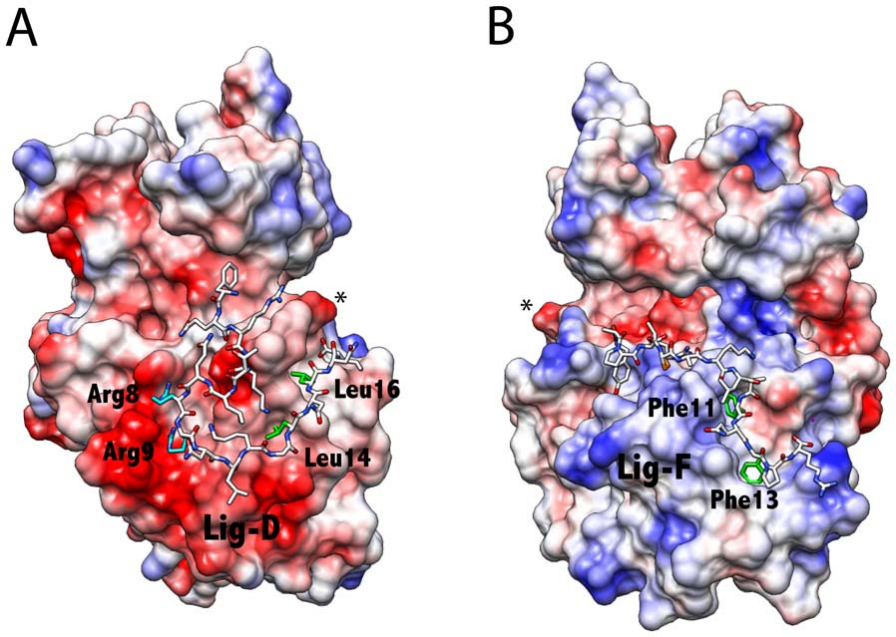
Molecular Models of Peptide Ligands bound to ERK2. **A**. Lig-F (YAPRAPAKLA-**FQFP**SR) bound to the FRS of ERK2. The Ψ-X-Ψ motif of Lig-F binds the FRS. **B**. Lig-D (FQRKTLQ**RR**NLKG**LNL**NL) bound to the DRS of ERK2. Residues important for binding are indicated. The Φ_A_ and Φ_B_ leucines of Lig-D occupy the hydrophobic Ø_1_ and Ø_2_ sites of ERK2. The conserved arginines bind the CD-domain.

To assess the importance of the interaction between the SAM domain of *Ets* and the FRS of ERK2 we designed a modular peptide ligand for the hydrophobic binding pocket of the FRS formed by the P+1 site, the αF helix, and the MAP kinase insert [9], [23] (Fig. 1A). This peptide called Lig-F contains an F-site, which is defined as ΨXΨP, where Ψ is an aromatic residue [26], [27], [28], [29]. To model the binding of Lig-F to the FRS, Leu-144–Leu153, Phe-183–Asn-199 and Phe-226–Leu-262 of ERK2 were set as the search regions for the FXFP binding motif of Lig-F. Compared to the docking model proposed by Lee et al. for a related F-site containing peptide, which seeks to accommodate all four residues of the motif into the hydrophobic pocket [23], our model shows the Phe residues buried deeply within the pocket, with the Pro more exposed to solvent (Fig. 4A). This mode of interaction is consistent with an earlier positional screening study, where little selectivity for proline was observed [9]. A comparison of the top 15 ranked structures, suggests that the *N*-terminus of Lig-F has conformational flexibility and can adopt a number of binding modes with the surface of ERK2.

**Figure 5:**
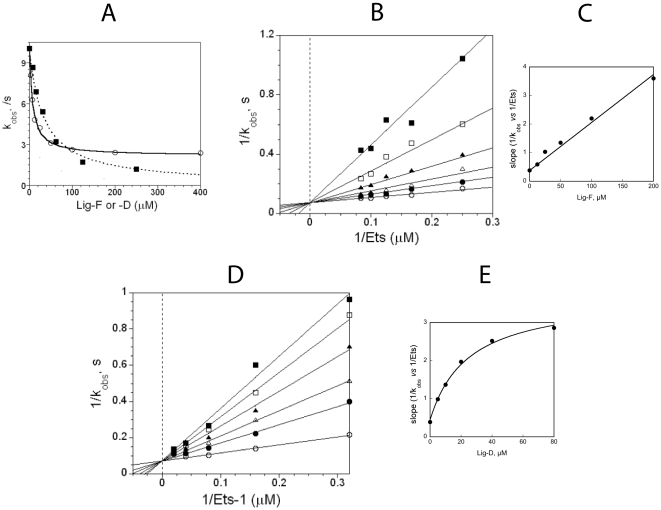
Inhibition of *Ets* phosphorylation. **A**) *k*_obs_ for the phosphorylation of *Ets* (6.4 µM) in the presence of Lig-D (0–400 µM, open circles) or Lig-F (0–400 µM, closed squares) and 1 mM MgATP. The line through the open circles (Lig-D, ) corresponds to the best fit to eqn. 6 for a dose-response curve to a partial competitive inhibitor where *K*_50_  = 7.3±0.8 µM, *k*_0_  = 8.0±0.2 s^−1^, and *k’*  = 2.1±0.1 s^−1^. The line through the closed squares (Lig-F, ▪) corresponds to the best fit to eqn. 5 for a dose-response curve for a full competitive inhibitor where *K*_50_  = 33.5±1.9 µM, and *k*_0_ = 10.3±0.2 s^−1^. **B**) Double reciprocal plot of 1/*k*_obs_ *vs* 1/[*Ets*] at varied fixed concentrations of Lig-F (0–200 µM) and 1 mM MgATP. Initial velocities were measured using various (2–12 µM) concentrations of *Ets*. The data were fitted to a model of competitive full inhibition according to eqn. 3 where 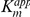 = 5.6±0.7 µM, 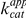  = 14.9±0.8 s^−1^, and 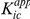  = 22±1.9 µM. **C**) Linear secondary plot of slope *vs* Lig-D for the plot in Fig. 5B. The line represents the best fit through the data according to the calculated parameters and the following equation; 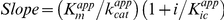 which is derived from eqn. 3. **D**) Double reciprocal plot of 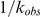 *vs* 1/[*Ets*] at varied fixed concentrations of Lig-D (0–80 µM) and 1 mM MgATP. Initial velocities were measured using various (3–59 µM) concentrations of *Ets*. The data were fitted to a model of partial competitive inhibition according to eqn. 4 where 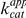  = 14.1±1.3 s^−1^, 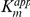  = 6.3±1.7 µM, 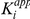  = 3.0±0.4 µM, 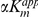  = 22±0.2, *α*  = 7.3±0.6 and *β* = 1. **E**) Non-linear secondary plot of slope *vs* [Lig-D] for the plot in Fig. 5D. The line represents the best fit through the data according to the calculated parameters and the following equation; 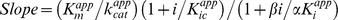 which is derived from eqn. 4.

To examine the ability of Lig-F to inhibit the phosphorylation of Ets-1 the effect of varying the concentration of *Ets* at several fixed concentrations of Lig-F was assessed. When the kinetic data were plotted in double reciprocal form a mechanism of competitive inhibition was revealed (Fig. 5B). Analysis of the secondary slope plot (Fig. 5C) shows the mechanism to be a linear competitive mechanism of inhibition. Thus, the kinetic experiment provides support for the model and the notion that the docking interaction at the FRS is critical for the formation of a complex between ERK2 and *Ets.*

### Exploring the catalytic function of docking at the DRS

The prediction that residues 10–16 of *Ets* engage the DRS of ERK2 is intriguing. To understand this further we sought to selectively compete out this interaction using an exogenous peptide. Previously, we have shown that a peptide that binds the DRS can displace *Ets* from ERK2 [11]. Our modeling suggests it occurs through competition with residues 10–16 of *Ets*, which presumably stabilize the complex. We decided to examine this further using a related peptide termed Lig-D, which we first modeled onto the surface of ERK2 using a molecular docking approach (Fig. 4B). The modeling of the binding of Lig-D to ERK2 was guided by the structure of its complex with the yeast MAP kinase Fus3 [18] as described early. In addition, distance restraints were employed, based on the known structures of several other D-site•MAPK complexes [22], [30], [31], [32], [33]. The top 15 ranked structures were found to conform to a similar binding mode, providing a high degree of confidence in the general locus and conformation. The top ranked structure is shown in Fig. 4B and supports the notion that it will compete with *Ets* for binding to the DRS of ERK2. A comparison of the structures suggests that the conformation of the *N*-terminal segment is not highly restricted in the complex and may adopt several conformations.

**Figure 6:**
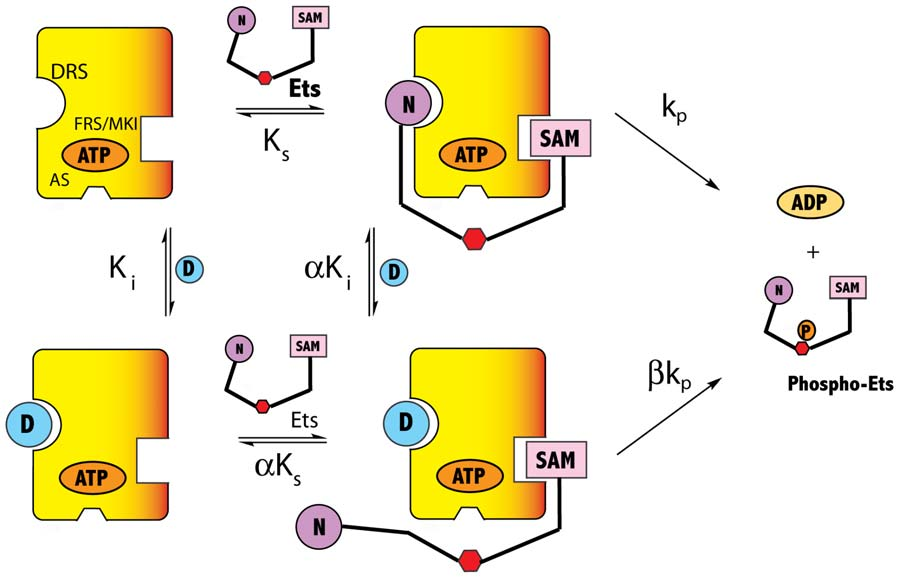
Mechanism of ERK2 Inhibition by Lig-D. Lig-D exhibits competitive partial inhibition of the phosphorylation of *Ets* by ERK2, which is described by eqn. 4 where *α* = 8 and *β* = 1.

Thus, based on the modeling Lig-D is predicted to block the binding of residues 10–16 of Ets-1 to the DRS of ERK2. To test this prediction kinetic studies were performed by varying the concentration of *Ets* at several fixed concentrations of Lig-D in the presence of 1 mM ATP. A double reciprocal plot of the data revealed a competitive mechanism of inhibition (See Fig. 5D) consistent with the notion that Lig-D competes with *Ets* for binding. However, an analysis of the secondary slope plot (Fig. 5E) revealed a hyperbolic mechanism of inhibition, suggesting that Lig-D does not completely inhibit the ability of ERK2 to phosphorylate *Ets*. The non-linear nature of the inhibition is further apparent when *k_obs_* (determined at a fixed concentration (6.4 µM) of *Ets*) is plotted against the concentration of Lig-D (0–400 µM) (Fig. 5A, open circles). As the concentration of Lig-D approaches 400 µM, *k_obs_* clearly decreases to a plateau corresponding to approximately 26% of its value in the absence of Lig-D. To understand this further the kinetic data were assessed in terms of the general mechanism for hyperbolic mixed inhibition (Fig. 6). The best fit to the data corresponds to a mechanism of competitive partial inhibition, which is described by eqn. 4 where *α*  = 8 and *β*  = 1. Thus, according to this mechanism Lig-D competes with Ets-1 for binding to ERK2, but it does not completely displace it. Surprisingly, while the ERK2•Lig-D complex is predicted to have a lower affinity for *Ets* compared to ERK2, the best fit to the kinetic data predicts that it phosphorylates *Ets* at an equal rate. This suggests that the docking interaction at the DRS plays no role in facilitating the phosphorylation of Thr-38 once the complex is formed.

**Figure 7:**
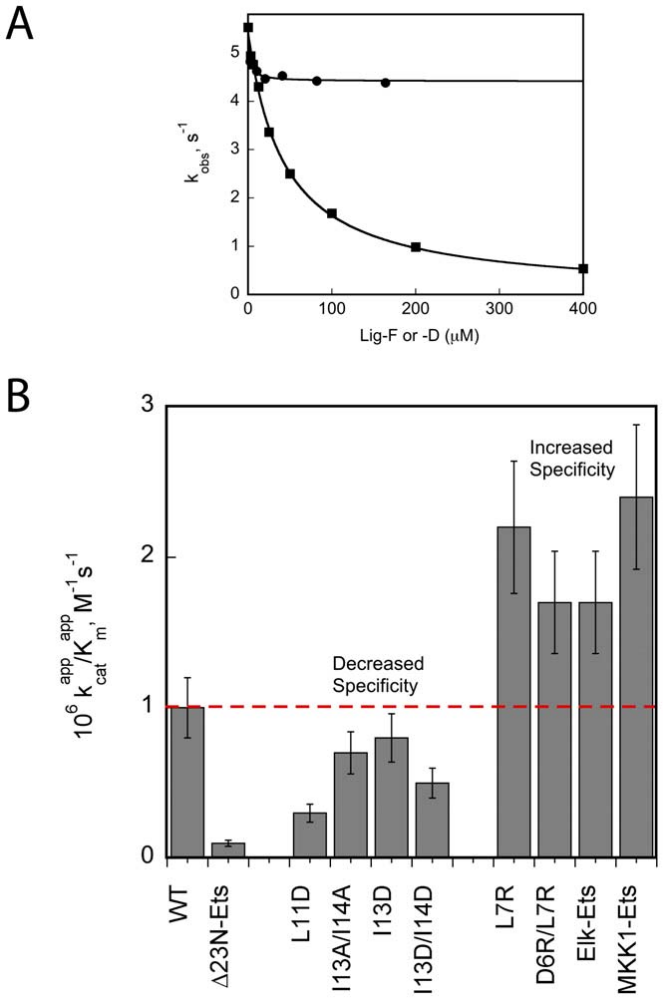
Inhibition of ERK2. **A**. *k*_obs_ for the phosphorylation of Δ23N-*Ets* (50 µM) in the presence of Lig-D (0–200 µM, (•) or Lig-F (0–400 µM, (▪) and 1 mM MgATP. The line through the circles (Lig-D) corresponds to the best fit to eqn. 6 for a dose-response curve to a partial competitive inhibitor where *K*_50_ = 2.0±0.5 µM, *k*_0_ = 5.5±0.1 s^−1^, *k’*  = 4.4±0.1 s^−1^. The line through the squares (Lig-F) corresponds to the best fit to a dose-response curve for a full competitive inhibitor (eqn. 5) where *K*_50_ = 43±2 µM, *k*_0_ = 5.4±0.1 s^−1^. **B**. Specificity constant, 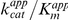
for the phosphorylation of *Ets* mutants compared to *Ets*.

The analysis above is intriguing and suggests that the *N*-terminus of *Ets* promotes the formation of the ERK2•*Ets* complex, but has a limited influence on *k*_cat_. Based on this observation we predicted that deletion of the docking site would lead to an enzyme-substrate complex that is weaker but undergoes phosphorylation at substantially the same rate as the ERK2•*Ets* complex. Thus, the first 23 amino acids were deleted from *Ets* to produce Δ23N-*Ets*. In line with our prediction ERK2 was found to phosphorylate Δ23N-*Ets* with a 14-fold higher *K*_m_ (Fig. 7A) but with a *k*_cat_ comparable to the *k*_cat_ for the phosphorylation of *Ets*. A further prediction of the model is that, unless induced conformational changes are important, Lig-D should have little influence on the phosphorylation of Δ23N-Ets. Indeed, as the model predicts Fig. 7A (•) shows that Lig-D exhibits little propensity to inhibit the phosphorylation of Δ23N-Ets.

Fig. 1C shows how a typical docking site (which contains the consensus (∑)_2-3_-X_2-6_-Φ_A_-X-Φ_B_ where Φ is an aliphatic hydrophobic and ∑ is basic) binds to the DRS of ERK2. A notable feature of the *N*-terminus of Ets-1 is that while it does not contain such a consensus sequence residues 10-16 contain a potential Φ_A_-X-Φ_B_ motif. Interestingly, the modeling placed Leu-11 and Ile-13 in the Φ_hyd_ subsite in the proximity of the Ø_1_ and Ø_2_ pockets respectively, suggesting that this motif may contribute to the association of the *N*-terminus of Ets-1 with ERK2. To further examine the model several mutations were introduced into the *N*-terminus of Ets-1 that were predicted to destabilize the interaction of the putative ^11^Φ_A_-X-Φ_B_^13^ motif. Specifically, the following mutants of *Ets*, Asp11, Asp13, Ala13/Ala14 and Asp13/Asp14 were prepared. The introduction of these mutations resulted in small yet significant effects on the steady-state kinetic parameters that are generally consistent with the notion that Leu-11 and Ile-13 (or Ile-14) interacts loosely with the DRS of ERK2 to promote binding (See Table 1). For example, substitution of Leu-11 for Asp resulted in a 3-fold increase in *K*_m_ with little effect on *k*_cat_. The double mutant I13D/I14D exhibited a similar increase in *K*_m_. Although these effects are subtle it should be noted that they correspond to a significant proportion of the total 10-fold increase in *K*_m_ that results from the deletion of the entire *N*-terminal 23 residues of *Ets*.

**Table 1:** Effect of mutations in the *N*-terminus of *Ets* on the steady-state parameters for *Ets* phosphorylation.

	Mutations in the *N*-terminus of *Ets*	*k*_cat_	*K*_m_	*k*_cat_/*K*_m_
	^1^XXXXXXXXXXΦXΦX^14^	s^−1^	µM	×10^6^ M^−1^s^−1^
*Ets*	^1^MKAAVDLKPTLTII^14^	18.0±2.0	17.0±4.0	1.0
D^11^	MKAAVDLKPTDTII	14.0±1.0	54±5.0	0.3
D^13^	MKAAVDLKPTLTDI	12.0±1.0	15.0±1.0	0.8
^13^AA^14^	MKAAVDLKPTLTAA	21.0±1.5	32.0±5.0	0.7
^13^DD^14^	MKAAVDLKPTLTDD	23.0±2.0	50.0±5.0	0.5
R^7^	MKAAVDRKPTLTII	13.0±1.0	5.0±1.0	2.2
^6^RR^7^	MKAAVRRKPTLTII	10.0±1.0	6.0±2.0	1.7
Elk-1 (312–324)	KGRKPRDLELPLS	11.0±1.0	7.0±2.0	1.7
MKK1 (1–13)	MPKKKPTPIQLN	12.0±0.3	5.0±0.6	2.4

In contrast to these mutations that led to a decrease in the specificity of the ERK2-*Ets* interaction, the modeling also allows for a prediction of potential mutants that might improve specificity. To this end an Arg residue was introduced in place of Leu-7 to create a R/K-(X)_2_-Φ_A_-X-Φ_B_ motif with potential to interact at the Φ_chg_ site. This mutant exhibits a 1.7-fold improved specificity (as determined by comparing *k*_cat_/*K*_m_) over *Ets,* which is largely due to the 3-fold decrease in *K*_m_. While the introduction of a second Arg at residue position 6 had no additional effect it was interesting to note that when the *N*-terminus was exchanged for the corresponding docking sites of Elk-1 and MKK1 (which conform to R/K-(X)_5_-Φ_A_-X-Φ_B_ and R/K-(X)_3_-Φ_A_-X-Φ_B_ consensus sequence respectively) the resulting proteins were also better substrate than *Ets* by a factor of approximately 2-fold. Taken together, the mutagenesis studies support the notion that the *N*-terminus of *Ets* serves as a weak ligand for the Φ_hyd_ site of the DRS of ERK2. A conclusion supported by the NMR studies (Fig. 3D).

## Discussion

MAPKs must overcome several challenges. For example, they need to rapidly find and recognize protein substrates in the cellular milieu, while discriminating against Ser/Thr residues located at incorrect positions on a protein, as well as on the wrong protein. Rather than view these as challenges for the MAPK alone however, it is reasonable to view them as challenges of an entire MAPK signaling network, especially the substrates. For example, because there are so many MAPK substrates of varied structure each one may be viewed as having to meet the challenge of evolving to adopt features that facilitate its efficient phosphorylation by a particular MAPK at one or more sites. For its part, each MAPK must evolve to be able to activate bound ATP, in such a manner that a suitably positioned hydroxyl group placed at the active site may be readily phosphorylated. The MAPK ERK2 possesses two sites called recruitment sites, the DRS and the FRS, which are frequently associated with substrate binding through what have been termed docking interactions (Fig. 1). Precisely how these recruitment sites facilitate the phosphorylation of a substrate has not been established, because no structure of a substrate bound to an active MAPK has been reported. Key questions regarding the mechanism of substrate phosphorylation address our understanding of how docking interactions control the rate of access of phosphorylation sites to the active site, what steps control and limit catalysis and what, if any, general features do substrates possess? To address these questions we previously studied the mechanism of phosphorylation of the transcription factor Ets-1, which is phosphorylated by ERK2 on Thr-38 exclusively [15]. Kinetic studies revealed an efficient mechanism of catalysis where both phosphorylation and product release are rate-limiting, with both phospho-*Ets* and ADP dissociating with similar rate constants [13], [15]. In addition, to the interesting kinetic characteristics of the catalytic reaction we discovered that *Ets* possesses two docking sites, which both contribute to the recognition of ERK2 [11] (Fig. 2).

To understand the molecular recognition in more detail we built the model shown in Fig. 3B, which was achieved using a combined molecular mechanics and docking approach. This model represents a conformation of the complex in which the Thr-Pro motif is primed for phosphorylation in the active site. Critically this model allows us to perform the first structure-function analysis of a MAPK-substrate interaction. The model suggests that both recruiting sites of ERK2 simultaneously participate in docking interactions, which may serve to restrain Thr-38 near to the active site. While the FRS is known to bind modular ΨXΨP motifs [9], the model predicts that it adopts a different mode of interaction with Ets-1, where instead it mediates a domain-domain interaction with the SAM domain of Ets-1. Thus, a region defined by Helix 4–loop–Helix 5 of the SAM domain is predicted to interact with the αG helix and α2L14 helix of ERK2 (Fig. 3B). A further feature of this model is that part of Helix 1 (residues 41-51) unwinds upon binding ERK2 (Fig. 3C), which may serve to allow Thr-38 to extend towards the active site of ERK2, as well as to facilitate the formation of a new binding interface. Recent studies suggest that mutation of both His-230 of ERK2 [11] and Phe-120 [14] of Ets-1, two residues predicted to be close to this interface, disrupt the stability of the complex. The model also suggests that residues 1–40 of Ets-1, which are intrinsically disordered in solution [21], remain predominantly disordered while bound to the surface of ERK2, with a short sequence corresponding to residues 10–16 engaging the hydrophobic groove of the DRS.

A careful analysis of the kinetic mechanism of inhibition by Lig-D reveals that it inhibits *Ets* phosphorylation through a mechanism of partial competitive inhibition (Fig. 6). A reasonable interpretation of the data is that Lig-D displaces the *N*-terminus of Ets-1 from the DRS of ERK2, thereby leading to a weakening of the complex. Alternatively, the binding of the peptide could promote a conformational change in ERK2 that leads to a weakening of the complex. We do not favor this latter explanation, however because the peptide exhibits little ability to displace the truncated form of *Ets*, Δ23N-*Ets* (Fig. 7A). Furthermore, a significant conformational change is not supported by the NMR experiments on inactive ERK2 (Piserchio et al. *submitted*). It is intriguing that despite the loss of the *N*-terminal interaction, *k*_cat_ for the phosphorylation of Δ23N-*Ets* is almost identical to that for the phosphorylation of *Ets*, suggesting that the tether does not significantly influence steps associated with turnover once the complex is formed. Consistent with this is the observation that mutations in the DRS have little influence on *k*_cat_, see Table 2 in Abramczyk et al. [12]_._

A notable feature of the *N*-terminus of Ets-1 is that while it does not contain a conventional consensus sequence for binding to the DRS of ERK2 it contains a potential Φ_A_-X-Φ_B_ motif. Interestingly, 4 out of the top 5 structures from 5 independent virtual docking runs suggested that Ile-13 of Ets-1 binds the Ø_2_ site of the DRS (Fig. 3A). Recently we completed a cysteine-foot-printing study that supported the notion that Ets-1 binds this pocket. We mutated several sites in the *N*-terminus, which were predicted by the modeling to either increase or decrease the specificity of *Ets* for ERK2. Through disruption of the putative Φ_A_-X-Φ_B_ site we were able to decrease the specificity of *Ets* for ERK2. Gratifyingly, by introducing a (RK)-X_2_-Φ_A_-X-Φ_B_ consensus sequence into the *N*-terminus we were able to slightly increase the specificity. Thus, ERK2 exhibits a 7-fold higher specificity for *Ets* L7R over *Ets* L11D. ERK2 is reported to phosphorylate Ets-2, which is related in sequence to Ets-1 [14]. Interestingly, while the SAM domains of both proteins share high sequence similarity the *N*-terminus of Ets-1 possesses little sequence similarity to the corresponding region of Ets-2 [14]. Thus, it will be interesting to examine both proteins to identify features that are common to both.

The modeling studies described here suggest that the *N*-terminus of Ets-1 remains essentially disordered upon binding to ERK2 and engages the DRS of ERK2. While the model shows Thr-38 bound to the active site we believe that given the disordered nature of the loop it may adopt several alternative conformations that are similar in energy. A binding study on a series of Thr-Pro mutants would appear to support the notion that the binding of the Thr-Pro motif does not contribute to the stability of the ERK2•*Ets* complex [5]. Thus the docking interactions identified by the modeling would appear to provide a topological constraint, which effectively restricts Thr-38 to the proximity of the active site. Such a mechanism of proximity-induced catalysis [5] would also allow for proximal sites to be phosphorylated within the same complex, without a requirement for a realignment of docking interactions.

Since the seminal paper by Wright [34] the role of intrinsically disordered regions of proteins has been a topic of great interest. For example, it has been suggested that on average intrinsically disordered proteins bind and dissociate 2-3 fold faster than ordered proteins [35]. There is some evidence to suggest that the recognition of MAPKs by proteins containing intrinsically disordered regions may be quite common. In fact, many docking sites for the DRS of a MAPK are found at the extreme termini of MAPK ligands, such as the upstream activators the MKKs, and may be disordered. For example, the *C*-terminus of the protein kinase MK2 contains a docking site for the DRS of its activator p38MAPKα [33], [36]. The structure of the complex between unactivated p38MAPKα and MK2 suggests that at least part of the region responsible for binding the DRS has a propensity to be disordered. We have used stopped-flow analysis to assess the rate of binding of phospho *Ets* to ERK2 and found that it binds with a rate that exceeds the rate of diffusion for the association of two proteins, with a rate constant for association of *k*_on_  = 5×10^6^ M^-1^s^-1^ and with rate constant for dissociation of *k* >100 s^-1^ [13]. It will be interesting to determine whether the disordered docking sites found at the extreme termini of signaling partners facilitate the rapid formation of signaling complexes within the MAPK pathways.

### Conclusion

The structural model of the ERK2•*Ets* complex suggests that Ets-1 simultaneously binds both the DRS and the FRS of ERK2 to position the phosphorylation site, Thr-38, in the proximity of the active site. Previous stopped-flow studies, which suggest that the association between ERK2 and *Ets* is both rapid and transient, raise intriguing questions about the role of the two docking sites. As cell signaling is a dynamic process, where proteins at low concentrations must often interact and dissociate rapidly and yet bind with high specificity, highly evolved protein-protein interactions are expected, to facilitate efficient recognition. The new model allows us to begin to examine the mechanism of binding as well as the conformational dynamics associated with phosphorylation once the enzyme substrate complex is formed. With further NMR studies on the horizon we hope to further refine the structure using a combination of computational and experimental approaches.

## Supporting Information

Table S1 **Distance constraints between Lig-D and ERK2 were set based on the known interactions of the two motifs (RR and Φ_A_-X-Φ_B_) with phosphatase (PDB ID: 2GPH).** Distance constraints between Lig-F and ERK2 were set to make the Phe-Xaa-Phe motif (DEF motif) binding a hydrophobic pocket formed between the P+1 site, the αF helix and the MAP kinase insert. The binding constraints for Ets(1-42) were imposed to enforce the hydrogen bonding interactions between Thr residue in the Thr-Pro motif with Asp-147 and Lys-149 of ERK2. In addition, the proline in the Thr-Pro motif was restrained to adopt a similar binding mode to that in Ser-Pro motif of HHASPRK bound to the cyclin-dependent kinase (CDK2) (PDB ID: 1QMZ).
(DOCX)
