# Supplementary material for: A Model of a MAPK•Substrate Complex in an Active Conformation: A Computational and Experimental Approach
Source: PLoS One. 2011 Apr 11;6(4):e18594. doi: 10.1371/journal.pone.0018594 (PMC3073974; doi:10.1371/journal.pone.0018594)
Supplement: Table S1 — Distance constraints between Lig-D and ERK2 were set based on the known interactions of the two motifs (RR and ΦA-X-ΦB) with phosphatase (PDB ID: 2GPH). Distance constraints between Lig-F and ERK2 were set to make the Phe-Xaa-Phe motif (DEF motif) binding a hydrophobic pocket formed between the P+1 site, the αF helix and the MAP kinase insert. The binding constraints for Ets(1-42) were imposed to enforce the hydrogen bonding interactions between Thr residue in the Thr-Pro motif with Asp-147 and Lys-149 of ERK2. In addition, the proline in the Thr-Pro motif was restrained to adopt a similar binding mode to that in Ser-Pro motif of HHASPRK bound to the cyclin-dependent kinase (CDK2) (PDB ID: 1QMZ). (DOCX) [file pone.0018594.s001.docx]

**Table S1**. **Distance constraints for Lig-D, Lig-F, and Ets(1-42).**

| Protein | | Ligand | | Distance | Spring Constant |
| --- | --- | --- | --- | --- | --- |
| Residue | Atom | Atom | Residue |  | (kJ·mol^-1^·Å^-2^) |
| **Lig-D** (^1^FQRKTLQ**RR**NLKG**LNL**NL^18^) | | | | | |
| CD site |  |  |  |  |  |
| Asp-319 | OD2 | NH2 | Arg-8 | 2.5-3.5Å | 5 |
| Asp-319 | OD2 | NE | Arg-8 | 2.5-3.5Å | 5 |
| Asp-319 | OD2 | NH2 | Arg-8 | 2.5-3.5Å | 5 |
| Glu-79 | OE1 | NH2 | Arg-8 | 2.5-3.5Å | 5 |
| Glu-79 | OE2 | NH1 | Arg-8 | 2.5-3.5Å | 5 |
| Tyr-129 | OH | NE | Arg-9 | 2.5-3.5Å | 5 |
| Tyr-129 | OH | NH2 | Arg-9 | 2.5-3.5Å |  |
| hydrophobic Ø_1_ and Ø_2_ sites | |  |  |  |  |
| Gln-117 | NE2 | O | Asn-15 | 2.0-4.0Å | 5 |
| Phe-127 | CZ | CG | Leu-16 | 2.0-4.0Å | 5 |
| Leu-155 | CB | CG | Leu-16 | 2.0-4.0Å | 5 |
| Cys-159 | SG | CG | Leu-16 | 2.0-4.0Å | 5 |
| His-123 | NE2 | CG | Leu-14 | 2.0-4.0Å | 5 |
| Tyr-126 | CB | CG | Leu-14 | 2.0-4.0Å | 5 |
| **Lig-F** (^1^YAPRAPAKLA**FQFP**SR^16^) | | | | | |
| Arg-192 | CB | CZ3 | Phe-11 | 1.5-3.5Å | 5 |
| Ile-196 | CB | CZ2 | Phe-11 | 1.5-3.5Å | 5 |
| Met-197 | SD | NE1 | Phe-11 | 1.5-3.5Å | 5 |
| Tyr-231 | CB | CB | Gln-12 | 1.5-3.5Å | 5 |
| Leu-232 | CG | CG | Gln-12 | 1.5-3.5Å | 5 |
| Leu-235 | CG | CE3 | Phe-13 | 1.5-3.5Å | 5 |
| Ala-258 | CA | CH2 | Phe-13 | 1.5-3.5Å | 5 |
| Leu-198 | CG | CZ3 | Phe-13 | 1.5-3.5Å | 5 |
| Tyr-261 | CG | CB | Pro-14 | 1.5-3.5Å | 5 |
| Tyr-261 | CB | CB | Pro-14 | 1.5-3.5Å | 5 |
| **Ets(1-42)** | | | | | |
| Tyr-185 | CD2 | CB | Pro-39 | 3.0-3.5Å | 4 |
| Tyr-185 | C | CB | Pro-39 | 2.5-2.8Å | 4 |
| Val-186 | CA | CG | Pro-39 | 3.7-4.0Å | 4 |
| Ala-187 | N | CG | Pro-39 | 3.5-3.6Å | 4 |
| Ala-187 | O | CG | Pro-39 | 2.5-3.0Å | 4 |
| Ala-187 | O | CD | Pro-39 | 2.9-3.1Å | 4 |
| Ala-187 | O | C | Thr-38 | 5.2-5.5Å | 8 |
| Ala-187 | N | N | Pro-39 | 5.2-5.5Å | 8 |
| Tyr-185 | O | CA | Pro-39 | 4.0-4.2Å | 8 |
| Tyr-185 | O | C | Pro-39 | 4.5-4.8Å | 8 |
| Tyr-185 | CB | N | Ser-40 | 6.2-6.5Å | 8 |
| Asp-147 | OD2 | OG1 | Thr-38 | 2.7-2.8Å | 8 |
| Lys-149 | NZ | OG1 | Thr-38 | 2.9-3.0Å | 8 |
| Thr-188 | OG1 | OG1 | Thr-38 | 3.7-3.9Å | 8 |
